# Bibliography

**Published:** 1901-06-15

**Authors:** 


P. Blakiston's Sons & Co. have just published the third edition of "Questions on Medical Subjects." It is a small book, three by four inches, but it contains 230 pages and asks 3,500 questions on all branches of medical science. It also, by figures, refers for the answers to the well-known Quiz Compends published by this house by page number. The questions are well selected and apparently cover the entire subject of medical study. Such things are usually not desirable, but this book contains no answers, it only raises the question and tells where its answer may be found. We have no doubt but a student's desire for answers to these questions will lead him to answer other questions of his own asking, should he faithfully make use of some such help as this to lead him into search for knowledge.

